# Ultrasonographic and anatomical examination of normal thyroid and internal parathyroid glands in goats

**DOI:** 10.1371/journal.pone.0233685

**Published:** 2020-05-29

**Authors:** Filip Pankowski, Sławomir Paśko, Joanna Bonecka, Olga Szaluś-Jordanow, Marcin Mickiewicz, Agata Moroz, Bartłomiej Jan Bartyzel

**Affiliations:** 1 Department of Morphological Sciences, Institute of Veterinary Medicine, Warsaw University of Life Sciences-SGGW, Warsaw, Poland; 2 Virtual Reality Techniques Division, Institute of Micromechanics and Photonics, Faculty of Mechatronics, Warsaw University of Technology, Warsaw, Poland; 3 Department of Small Animal Diseases with Clinic, Institute of Veterinary Medicine, Warsaw University of Life Sciences-SGGW, Warsaw, Poland; 4 Division of Veterinary Epidemiology and Economics, Institute of Veterinary Medicine, Warsaw University of Life Sciences-SGGW, Warsaw, Poland; University of Glasgow, UNITED KINGDOM

## Abstract

Ultrasonographic examination of the normal thyroid and parathyroid glands has been described for humans and many animal species. However, similar reports for goats are still missing. The aim of the study was to present ultrasound features of the normal thyroid and internal parathyroid glands in goats with the determination of their dimensions and volume, followed by a comparison of the results to the gross examination. Seventy-two goats were used in the study. The echostructure and echogenicity of the thyroid and parathyroid glands were assessed. The length, width and height of the thyroid and the length and width of the parathyroid glands were measured. The thyroid volume was calculated using the ellipsoid formula, basing on the ultrasonographic dimensions. Size and volume of the dissected thyroid glands were established grossly, followed by a histological examination. In order to accurately describe the anatomy of the thyroid, new anatomical terminology characterizing this gland was proposed. The mean dimensions of the thyroid lobes were 30.2 x 10.5 x 6.3 mm. There were no statistically significant differences between the right and left lobe. Parathyroid glands measured an average of 3.6 x 2.4 mm. The percentage Root Mean Square Error between the results of ultrasonographic and gross examination was 16.73%, 20.65% and 17.01% for thyroid length, width and height, respectively, and 46.30% for volume. In order to obtain more precise calculation of the thyroid volume, a modified correction factor for the ellipsoid formula was introduced. For the first time, the normal ultrasonographic characteristics and dimensions of the caprine thyroid and internal parathyroid glands were presented. The results may serve as a radiological reference and be the basis for further research.

## Introduction

Thyroid and parathyroid ultrasound (US) is an internationally recognized diagnostic modality. In human medicine, it plays a major role in detecting and monitoring focal lesions or performing ultrasound-guided biopsy, as well as when diffuse thyroid disease is suspected [1,2]. Among domestic animals, thyroid US is commonly performed in dogs with suspected primary hypothyroidism or thyroid cancer [3] and in cats with hyperthyroidism [4]. In these companion animals, ultrasonographic anatomy and features of the diseases of the aforementioned glands are widely described [5–7]. Sonographic examination of the normal thyroid was also reported in cattle, horses and even dolphins [8–10].

However, there is a lack of similar reports for domestic goat (*Capra hircus*). In this species, US is widely used in reproduction, because it’s a fast, cheap, non-invasive and very effective method providing information directly affecting herd management [11]. The versatility of this modality, increasing equipment quality and its greater availability result in its growing application in goat medicine. It can be used to examine the abdominal cavity, especially gastrointestinal and urinary tract, parenchymal organs, major blood vessels and greater omentum, as well as the heart [12–15]. An US can even be used to confirm the presence of parasitic cysts in the brain in the course of cenurosis [16].

The goat thyroid consists of two spindle-shaped lobes located on each dorsolateral surface of the cranial trachea. The lobes are connected by a poorly developed fibrous isthmus. This gland has a slightly different shape in different species of domestic animals [17]. In humans, it is shaped in the letter H or U, has a marked glandular isthmus and may have an additional pyramidal lobe [18]. Parathyroid glands in both humans and animals are divided into two pairs, so-called internal and external. Each thyroid lobe has one of a pair of internal parathyroid glands embedded in its parenchyma. The external parathyroid glands have a variable location, they can be situated closely to the thyroid gland, near the bifurcation of the common carotid artery, ventral to the wing of the atlas or be embedded in the mandibular salivary gland [19].

Pathological conditions of the thyroid, such as atrophy, fibrosis, diffuse hyperplasia, cystic hyperplasia and neoplasia have been described in goats [20,21]. Goiter (thyroid gland enlargement), being an expression of the hypothyroid state, is still of great importance in this species. Nutritional deficiency of iodine, resulting from the deficit of this element in the environment, primarily soil, or consumption of plants containing goitrogenic substances are considered the main reasons [22]. Genetic causes, such as thyroglobulin synthesis defect transmitted in an autosomal recessive manner, have also been described [23,24].

Goats are increasingly being kept as companions, especially in developed countries. Owing to the owners’ emotional attachment to the individuals and care for their health, advanced medical care is expected, which so far was intended for traditional pet species like dogs and cats. There is a growing need in veterinary medicine to reach for normal radiological anatomy for subsequent species of animals, such as goats.

Therefore, the aim of this study was to present US features of normal thyroid and internal parathyroid glands in goats, determining their dimensions and volume and relating them to gross measurements, as well as to describe the technical aspects of US examination of these glands.

## Materials and methods

This study was carried out on cadavers; thus, according to the Polish legal regulations, the approval by Local Ethics Committee for Animal Experiments was not required.

### Animals

Seventy-two adult female goats aged 1.6–11.3 years, belonging to Polish White Improved and Polish Fawn Improved breeds were used. The animals came from a dairy herd and were intended for culling due to the clinical form of caprine arthritis-encephalitis (CAE), confirmed by ELISA (ID Screen MVV/CAEV Indirect Screening test, ID.vet; Innovative Diagnostics, Grabels, France). A catheter was placed in the cephalic vein and a mixture of xylazine (Xylapan, Vetoquinol, Poland) at a dose of 0.05 mg/kg and ketamine (VetaKetam, VetAgro, Poland) at a dose of 10 mg/kg was given intravenously. The animals were euthanized under general anesthesia by overdose of pentobarbital (Morbital, Biowet Puławy, Poland) at a dose of 30 mg/kg, given intravenously. There were no clinical signs of thyroid or parathyroid glands disease in the animals included in the study. Initially, 90 goats were enrolled in the study, but 18 were excluded due to thyroid lesions visible in US examination or during necropsy.

### US technique and image analysis

**Fig 1:**
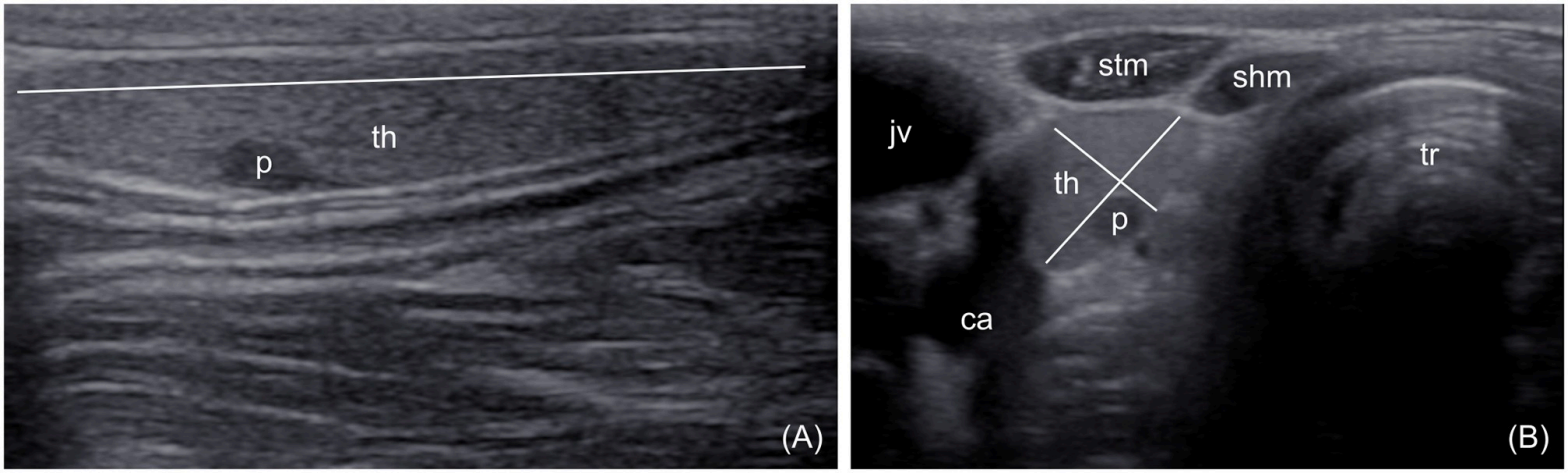
Ultrasonographic image of the thyroid lobe. (A) Longitudinal plane. (B) Transverse plane. The lines show how the maximum length, width and height of the thyroid lobe were obtained. p–parathyroid, th–thyroid, jv–jugular vein, ca–carotid artery, tr–trachea, stm–sternothyroid muscle, shm–sternohyoid muscle.

US examination was performed on the cadavers immediately after euthanasia. The animals were positioned in lateral recumbency with head and neck straight. The ventral cervical region was clipped and alcohol and coupling gel were applied to the skin. A medical ultrasound unit (HM70A, Samsung Electronics Ltd., UK) with a blended frequency 5–13 MHz linear transducer was used, using the highest possible frequency that allowed imaging of the entire thyroid gland. Each lobe was scanned in B-mode in longitudinal and transverse imaging planes. US images of the lobes were evaluated, especially echostructure and echogenicity of the parenchyma (comparing to the sternothyroid muscle), shape and borders of the gland. The maximum thyroid length (*l*_*t*_) in longitudinal plane and thyroid width (*w*_*t*_) and height (*h*_*t*_) in transverse plane were measured, as shown in Fig 1. The thyroid volume (*v*_*t*_) was then calculated using the ellipsoid formula (Eq 1):

$$v_{t}=l_{t}w_{t}h_{t}\frac{\pi }{6}$$ (1)

Internal parathyroid glands were assessed similarly, determining their shape, echostructure, echogenicity and location relative to the thyroid parenchyma. The measurements of the maximum parathyroid length (*l*_*p*_) and width (*w*_*p*_) were performed in longitudinal plane. US images were saved, exported and analyzed in the DICOM viewer (Horos, Nimble Co LLC d/ b/a Purview, Annapolis, MD, USA).

### Gross anatomy examination

The thyroid gland was dissected, removed and thoroughly cleaned from adjacent soft tissues immediately after US examination. The presence and appearance of an isthmus were assessed, after which both lobes were separated from each other and the isthmus was removed. Macroscopic look and shape were determined for each lobe. Lobes were weighed independently using an electronic balance (Axis AD2000, precision 0.01 g) and measured using an electronic caliper (TESA CAL IP67, precision 0.01 mm), analogously to measurements obtained in US. Volumes of thyroid lobes were measured using the method of water displacement. They were placed in a volumetric flask (precision 0.1 ml) filled with water at room temperature and the volume of displaced water was evaluated. The presence and appearance of internal parathyroid glands were assessed.

### Histologic examination

Thyroid and internal parathyroid glands were subjected to histologic examination. The glands were immersed in 10% buffered formalin for a week, dehydrated, embedded in paraffin, sectioned at 5-μm and stained with hematoxylin and eosin.

### Statistical analysis

Distributions of parameters measured as part of gross anatomy examination as well as those made using US were analyzed using the integrated software set R in version 3.6.0. These analyzes were carried out in two ways, i.e. independently for the right and left lobes, and for the entire data without the differentiation between sides. The functions used to calculate some distribution parameters, such as the minimum, maximum, mean and standard deviations values were taken from the „base” package in version 3.6.1. The median value was determined using the function from the "stats" package in version 3.6.1. Functions implementing Pearson’s and Spearman’s correlations were part of the Hmisc version 4.3–0 package, while Kendal correlation came from the "stats" package. In case when both compared parameters were characterized by a normal distribution, Pearson’s correlation was calculated, otherwise Spearman’s. The similarity of the categorization data was checked by the Pearson’s Chi-squared Test for Count Data, and the continuous by two-sided Wilcoxon Rank Sum and Signed Rank Tests. Both functions were part of the "stats" package. The function that allowed the Shapiro-Wilk test to be performed and the one-way ANOVA test function came from the same package, which made it possible to assess the correlation between the continuous and categorization variables.

## Results

In order to more precisely describe the anatomy of the thyroid gland and due to the lack of comprehensive nomenclature in Nomina Anatomica Veterinaria (N.A.V.) [25], new terminology was proposed in accordance with the guidelines outlined in N.A.V. The most cranial and caudal parts of the gland were called cranial extremity (*extremitas cranialis*) and caudal extremity (*extremitas caudalis)*, respectively. The surface directed towards the trachea was called medial surface (*facies medialis*) and the one directed towards the side of the neck was called lateral surface (*facies lateralis*). The margins directed towards the ventral and dorsal part of the neck were called ventral margin (*margo ventralis*) and dorsal margin (*margo dorsalis*), respectively.

In US, the thyroid gland was perfectly visible in all goats due to its size, superficial location and easy access. Thyroid lobes were of homogeneous echostructure and echogenicity, higher than that of the sternothyroid muscle. The margins were smooth and surrounded by a hyperechoic capsule (Fig 1). A hyperechoic hilum with blood vessels was variably visible in the cranial half of the lobe at the medial surface. In the long axis, the lobes had a rounded cranial margin and a narrowed to rounded caudal margin. In the short axis, the shape was usually semi-oval, with flattening of the medial surface. The mean US dimensions of the thyroid lobes were 30.2 mm in length, 10.5 mm in width, 6.3 mm in height and 1.06 cm^3^ in volume, using Eq 1 (Table 1). Internal parathyroid glands were seen as a round, homogeneously hypoechogenic, well marginated area (Fig 1). They were visible in 118/144 lobes (82%). The mean dimensions were 3.6 mm and 2.4 mm in the long and short axes, respectively (Table 1). A list of dimensions obtained in the US examination for all goats is available in the S1 Table.

**Table 1:** Ultrasonographic dimensions of the thyroid and parathyroid glands.

With the differentiation into the right and left lobe										
	*l*_*t*_ [mm]		*w*_*t*_ [mm]		*h*_*t*_ [mm]		*l*_*p*_ [mm]		*w*_*p*_ [mm]	
	R	L	R	L	R	L	R	L	R	L
min	19.40	18.00	7.00	7.00	4.10	4.30	1.90	2.00	1.30	1.50
max	36.60	37.60	14.90	15.50	9.60	8.50	5.60	5.30	3.60	3.60
range	17.20	19.60	7.90	8.50	5.50	4.20	3.70	3.30	2.30	2.10
mean	30.23	30.19	10.57	10.43	6.34	6.18	3.64	3.61	2.40	2.37
median	30.50	30.00	10.60	10.10	6.35	6.10	3.70	3.70	2.30	2.40
SD	3.73	3.76	1.67	1.66	1.21	1.08	0.89	0.82	0.54	0.49
p	0.86		0.49		0.49		0.91		0.88	
Without the differentiation into the right and left lobe										
	*l*_*t*_ [mm]		*w*_*t*_ [mm]		*h*_*t*_ [mm]		*l*_*p*_ [mm]		*w*_*p*_ [mm]	
min	18.00		7.00		4.10		1.90		1.30	
max	37.60		15.50		9.60		5.60		3.60	
range	19.60		8.50		5.50		3.70		2.30	
mean	30.21		10.50		6.26		3.62		2.39	
median	30.00		10.40		6.30		3.70		2.35	
SD	3.73		1.66		1.15		0.86		0.52	

*l*_*t*_−thyroid length, *w*_*t*_−thyroid width, *h*_*t*_*−*thyroid height, *l*_*p*_−parathyroid length, *w*_*p*_−parathyroid width, R–right lobe, L–left lobe, SD–standard deviation, p–significance level (Wilcoxon Rank Sum Test).

**Fig 2:**
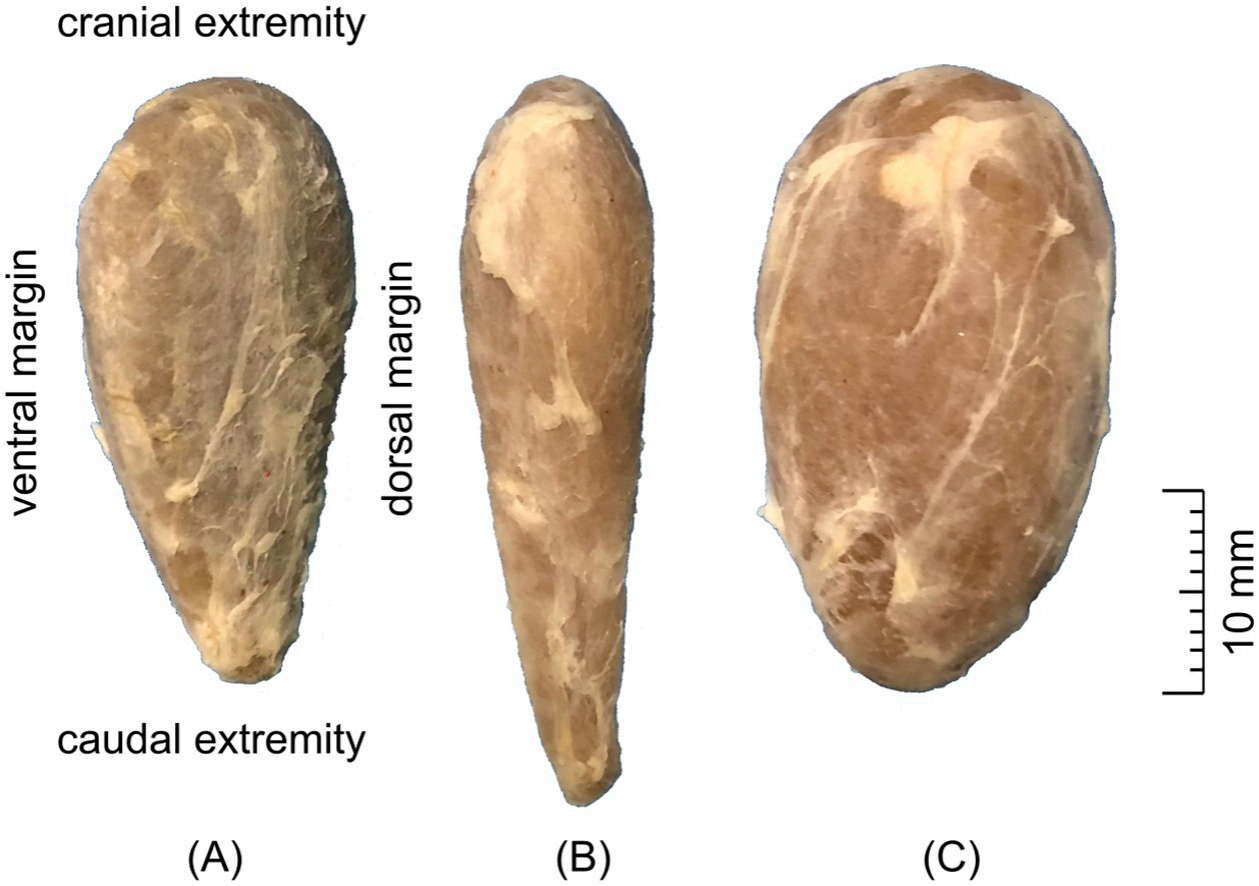
Gross photograph of the lateral surface of the right thyroid lobe. Three different types of shape are shown–(A) pear-shaped, (B) spindle-shaped, (C) oval.

**Fig 3:**
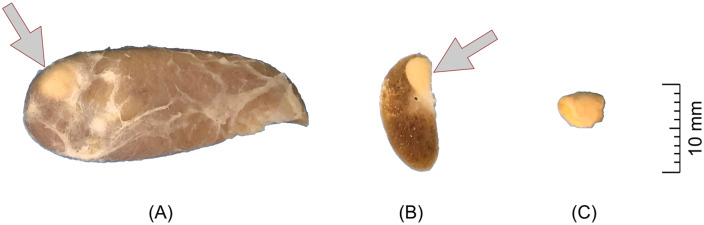
Gross photograph of the internal parathyroid gland. (A) medial surface view and (B) transverse section view of the parathyroid gland embedded in the thyroid lobe. (C) The same parathyroid gland after dissection from the thyroid gland.

In gross examination, the thyroid gland was moderately solid, yellowish brown. The lobes were categorized based on the macroscopic morphology into 3 types of shape: pear-shaped, fusiform, oval (Fig 2). The most common type, i.e. pear-shaped (73% of cases), was characterized by a rounded cranial margin and narrowed caudal margin, convex lateral surface and flat medial surface, as well as a slightly convex ventral and dorsal margins. The fusiform type (18% of cases) had significantly greater length over other dimensions. The oval type (9% of cases) had rounded both cranial and caudal margins at a relatively small length. The mean gross dimensions of the thyroid lobes were 32 mm in length, 12.6 mm in width and 6.8 mm in height and 1.6 cm^3^ in volume (Table 2). A thin, poorly developed fibrous isthmus was visible in the form of a light band of soft tissue connecting caudal extremities of both lobes. The internal parathyroid glands were visible at the medial surface of 104/144 (72%) thyroid lobes. They were most often located in their cranial half and had the appearance of a well-marginated, round, bright yellow area (Fig 3). A list of dimensions obtained in the gross examination for all goats is available in the S2 Table. Thyroid and internal parathyroid glands were histologically normal.

**Table 2:** Gross anatomical dimensions of the thyroid gland.

With the differentiation into the right and left lobe										
	*l*_*t*_ [mm]		*w*_*t*_ [mm]		*h*_*t*_ [mm]		*v*_*t*_[cm^3^]		*wt*. [g]	
	R	L	R	L	R	L	R	L	R	L
min	19.50	19.60	9.00	7.70	3.90	4.20	0.60	0.50	0.58	0.56
max	47.70	48.50	17.50	18.30	10.00	9.30	3.60	4.00	3.93	4.00
range	28.20	28.90	8.50	10.60	6.10	5.10	3.00	3.50	3.35	3.44
mean	31.89	32.05	12.66	12.51	6.83	6.82	1.62	1.66	1.75	1.74
median	31.45	31.50	12.50	12.50	6.95	6.90	1.50	1.60	1.59	1.68
SD	5.45	6.25	2.10	2.04	1.24	1.17	0.69	0.70	0.71	0.68
p	0.88		0.90		0.77		0.80		0.99	
Without the differentiation into the right and left lobe										
	*l*_*t*_ [mm]		*w*_*t*_ [mm]		*h*_*t*_ [mm]		*v*_*t*_ [cm^3^]		*wt*. [g]	
min	19.50		7.70		3.90		0.50		0.56	
max	48.50		18.30		10.00		4.00		4.00	
range	29.00		10.60		6.10		3.50		3.44	
mean	31.97		12.59		6.82		1.64		1.75	
median	31.50		12.50		6.90		1.50		1.67	
SD	5.84		2.07		1.20		0.69		0.69	

*v*_*t*_*−*thyroid volume, *wt*.*–*thyroid weight, for the rest of the legend see Table 1.

There was no statistically significant difference between the right and left thyroid lobe. The values of selected correlations of ultrasonographically obtained parameters of the thyroid and parathyroid glands are presented in Table 3 and of grossly obtained parameters in Table 4. Depending on the distribution of parameters for thyroid and parathyroid glands, they were correlated using Pearson’s or Spearman’s correlations. Parameters from the US examination with differentiation into the right and left lobe showed a lack of correlation between the width and height of the thyroid gland and the length and width of the parathyroid gland. A similar lack of correlation occurred when the sides were not differentiated. In addition, for the right side there was no correlation between thyroid length and parathyroid width (p = 0.06). The remaining parameters correlated for both US and gross examinations.

**Table 3:** Correlation results between ultrasonographically measured parameters of the thyroid and parathyroid glands.

	Right lobe				
	*l*_*t*_	*w*_*t*_	*h*_*t*_	*l*_*p*_	*w*_*p*_
*l*_*t*_	-	**r = 0.35**	**r = 0.29**	**r = 0.26**	r = 0.24
		**p = 0.003**	**p = 0.013**	**p = 0.04**	p = 0.06
*w*_*t*_	**r = 0.33**	-	**r = 0.63**	r = 0.13	r = 0.18
	**p 5.0e-3**		**p = 2.6e-9**	p = 0.29	p = 0.16
*h*_*t*_	**r = 0.39**	**r = 0.59**	-	r = -0.11	r = 0.03
	**p = 7.35e-4**	**p = 6.12e-08**		p = 0.38	p = 0.82
*l*_*p*_	**r = 0.45**	r = 0.079	r = 0.249	-	**r = 0.87**
	**p = 5.95e-4**	p = 0.56	p = 0.067		**p = 2.2e-16**
*w*_*p*_	**r = 0.40**	r = 0.099	r = 0.245	**r = 0.868**	-
	**p = 2.44e-3**	p = 0.47	p = 0.071	**p = 2.2e-16**	
	*l*_*t*_	*w*_*t*_	*h*_*t*_	*l*_*p*_	*w*_*p*_
	Left lobe				
Without the differentiation into the right and left lobe					
	*l*_*t*_	*w*_*t*_	*h*_*t*_	*l*_*p*_	*w*_*p*_
*l*_*t*_	-	**r = 0.33**	**r = 0.34**	**r = 0.32**	**r = 0.28**
		**p = 6.1e-05**	**p = 3.45e-05**	**p = 8.9e-4**	**p = 2.62e-3**
*w*_*t*_	-	-	**r = 0.60**	r = 0.067	r = 0.044
			**p = 2.65e-15**	p = 0.49	p = 0.65
*h*_*t*_	-	-	-	r = 0.033	r = 0.083
				p = 0.73	p = 0.39
*l*_*p*_	-	-	-	-	**r = 0.86**
					**p = 2.2e-16**
*w*_*p*_	-	-	-	-	-

The occurrence of correlation between the compared parameters is marked in bold. r–correlation coefficient, for the rest of the legend see Table 1.

**Table 4:** Correlation results between grossly measured parameters of the thyroid gland.

	Right lobe				
	*l*_*t*_	*v*_*t*_	*w*_*t*_	*h*_*t*_	*wt*.
*l*_*t*_	-	**r = 0.69**	**r = 0.43**	**r = 0.30**	**r = 0.64**
		**p = 6.7e-09**	**p = 1.7e-4**	**p = 0.01**	**p = 2.9e-09**
*v*_*t*_	**r = 0.72**	-	**r = 0.86**	**r = 0.86**	**r = 0.99**
	**p = 2.1e-09**		**p < 2.2e-16**	**p = 2.8e-16**	**p < 2.2e-16**
*w*_*t*_	**r = 0.42**	**r = 0.89**	-	**r = 0.76**	**r = 0.86**
	**p = 2.5e-4**	**p < 2.2e-16**		**p = 7.4e-15**	**p < 2.2e-16**
*h*_*t*_	**r = 0.28**	**r = 0.76**	**r = 0.74**	-	**r = 0.80**
	**p = 1.8e-2**	**p = 5.9e-11**	**p = 2.2e-13**		**p < 2.2e-16**
*wt*.	**r = 0.71**	**r = 0.98**	**r = 0.82**	**r = 0.77**	-
	**p = 1.2e-11**	**p < 2.2e-16**	**p = 2.2e-16**	**p = 7.7e-15**	
	*l*_*t*_	*v*_*t*_	*w*_*t*_	*h*_*t*_	*wt*.
	Left lobe				
Without the differentiation into the right and left lobe					
	*l*_*t*_	*v*_*t*_	*w*_*t*_	*h*_*t*_	*wt*.
*l*_*t*_	-	**r = 0.71**	**r = 0.38**	**r = 0.27**	**r = 0.66**
		**p < 2.2e-16**	**p = 2.5e-06**	**p = 9.8e-4**	**p = 2.2e-16**
*v*_*t*_	-	-	**r = 0.87**	**r = 0.81**	**r = 0.99**
			**p < 2.2e-16**	**p < 2.2e-16**	**p = 2.2e-16**
*w*_*t*_	-	-	-	**r = 0.75**	**r = 0.84**
				**p < 2.2e-16**	**p < 2.2e-16**
*h*_*t*_	-	-	-	-	**r = 0.79**
					**p < 2.2e-16**
*wt*.	-	-		-	-

The occurrence of correlation between the compared parameters is marked in bold. For the legend see Tables 1, 2 and 3.

For gross measurements at a confidence level of 0.05, the pear-shaped and fusiform shapes differed statistically only in length. The oval shape differed from the above-mentioned in all the other parameters, except the height when compared to the pear-shaped. For ultrasonographic measurements, only the fusiform and oval shapes were statistically different by width.

There was a weak correlation between the age of goats and thyroid length (r = 0.20, p = 0.026) and thyroid width (r = 0.23, p = 0.009). The age did not correlate with any other parameter.

The length, width and height of the thyroid gland measured grossly correlated with the same parameters measured ultrasonographically. There was a difference between them, which was reflected by the value of Root Mean Square Error (RMSE) (Table 5).

**Table 5:** Correlation results between ultrasonographically and grossly obtained thyroid parameters.

*l*_*t*_	*w*_*t*_	*h*_*t*_	*v*_*t*_
r = 0.53	r = 0.62	r = 0.65	r = 0.72
p = 1.4e-11	p = 2.6e-16	p = 2.2e-16	p < 2.2e-16
RMSE = 5.35 [mm]	RMSE = 2.6 [mm]	RMSE = 1.16 [mm]	RMSE = 0.76 [cm^3^]

RMSE–Root Mean Square Error, for the rest of the legend see Tables 1 and 3.

It was assumed that the reference values in that comparison were these obtained grossly. The RMSE was then related to the mean volume of the reference and the following values were received: 16.73% for length, 20.65% for width, 17.01% for height and 46.30% for volume.

Based on the values of mean distribution of gross and US volume, a correction factor equal to the quotient of these values was obtained. The result was 1.53, i.e. approximately $\frac{3}{2}$. It was entered into Eq 1, and the Eq 2 was introduced:

$$v_{t}=l_{t}w_{t}h_{t}\frac{\pi }{4}$$ (2)

Using Eq 2, a decrease in RMSE to the value of 0.51 [cm^3^] was obtained for the US volume, which in relation to the reference value gives 31.01%. The mean volume calculated by the Eq 2 was 1.62 ± 0.61cm^3^.

## Discussion

To the best of authors’ knowledge, this is the first study presenting the normal US images and dimensions of the thyroid and internal parathyroid glands in goats, thus filling the missing gap in the knowledge of diagnostic imaging of these glands in domestic animals.

The largest difference between US and gross dimensions in the current study was in the length of the thyroid gland. Taeymans et al. [26] found that the greatest intra- and interobserver variability of US measurements of the thyroid gland in Beagle dogs was related to this dimension as well. This was explained by the difficulty in locating the caudal extremity of the gland caused by the variable shape of the lobe and therefore the difficulty in obtaining its ideal long axis. A similar conclusion can be drawn in studies on goats. The maximum length of the thyroid gland in the current study (37.6 mm) was even higher than in the mentioned studies (28.5 mm), which may indicate a greater likelihood of underestimation (the longer the structure, the more challenging it is to obtain a single scan over its entire length). Another possible explanation given in the above work was the risk of measuring external parathyroid gland, which didn’t differ ultrasonographically from the thyroid parenchyma, together with the thyroid gland and thus falsely overstating its length. This phenomenon didn’t apply to goats, because parathyroid glands were clearly distinguishable from the thyroid.

For calculation of the thyroid volume based on the maximum length, width and height, the ellipsoid formula (Eq 1) is commonly used. The correction factor in that equation is $\frac{\pi }{6}$ (= 0.524). In 1981, Brunn et al. proposed a modified correction factor of 0.479, as more precise compared to the previous one [27]. In 2006, Shabana et al. compared the results of the US volume measured using different correction factors with the results of the volume obtained in the computed tomography and proposed another factor of 0.529, as an average of the acceptable range 0.494–0.554 [28]. In the current study, the correction factor of $\frac{\pi }{6}$ was initially used, as widely accepted in human and veterinary medicine. However, due to the discrepancy between the results of US and gross volume, a modified correction factor of $\frac{\pi }{4}$ (= 0.785) was introduced. The volume calculated this way (Eq 2) was 1.62 ± 0.61 cm^3^, which is similar to the grossly measured 1.64 ± 0.69 cm^3^. For comparison, the mean volume calculated using the general formula (Eq 1) was 1.06 ± 0.40 cm^3^. The cause of this disparity was presumably the fact that only 9% of caprine thyroids were oval-shaped, i.e. similar to an ellipsoid, thus fitted well to the general ellipsoid formula. For other thyroid shapes this formula turned out to be much less precise.

US examination was not reliable when categorizing thyroid lobes to the shape type, as was done in the gross examination. To reflect the true shape of an organ it is necessary to obtain a scan in the appropriate plane, which was often technically difficult and inconclusive. It is known that there is a risk of displacement and slight deformation of the thyroid due to its compression by an US transducer or adjacent anatomical structures, as well as the animals’ movement during the examination [29].

Normal human parathyroid glands measure approximately 6 mm in the craniocaudal dimension and 3–4 mm in the transverse dimension and are rarely identified in US [30]. According to Liles et al., canine parathyroid glands have a mean size of 3.3 x 2.2 x 1.7 mm and are aechogenic or hypoechogenic compared to thyroid tissue [31]. Woods et al. found that feline parathyroid glands in ultrasound are often invisible and establishing their reference size is inconclusive [32]. In humans and both mentioned species it is also difficult to distinguish parathyroid glands from thyroid lesions. In addition, the location of the parathyroid glands in cats was variable, so it couldn’t be assessed in advance in which part of the thyroid lobe they should be sought. In the vast majority of the thyroids examined in the current study (116/144 lobes; 81%), the internal parathyroid glands were identified, and they were located in the part of the thyroid they were expected to be found (i.e. cranial half, medial surface). As authors above reported for other species, the parathyroid glands were hypoechogenic also in goats. Therefore, it seems that the ambiguity in distinguishing between parathyroid glands and thyroid focal lesions described in humans, dogs and cats is much less expressed in goats. Compared to cats, the main reason of this discrepancy could be sought in the much larger size of the caprine parathyroid gland, and thus its greater visibility. Compared to dogs and humans, the size of the parathyroid glands did not differ significantly, hence one of the reasons may be their more constant occurrence and location in goats. Perhaps this is due to smaller breed differences within the species. However, it should be borne in mind that all the studied animals belonged to two breeds of comparable size, which could have resulted in greater consistency of obtained results.

There are no reports on US features of thyroid and parathyroid glands diseases in goats. In humans, the goiter is characterized by enlargement of the gland, rounding of the margins and sometimes irregular echotexture. In the congenital goiter, greater development and increased visibility of the isthmus is additionally described [33]. In dogs with hypothyroidism, the glands can have decreased echogenicity and smaller size [34]. In goats, as in other species, US may be of great importance in the diagnosis of thyroid diseases.

The congenital goiter in goat kids is manifested by severe symptoms and usually can be easily diagnosed in clinical examination. However, subclinical hypothyroidism may remain undiagnosed due to weak or non-specific clinical signs. It has a negative impact on production efficiency, growth and fertility in affected animals [35]. In such cases, the thyroid gland may be of normal size or only slightly enlarged. Therefore, US assessment of the thyroid size and parenchyma and comparison to reference values could aid in diagnosis.

Thyroid US is used in public health in underdeveloped countries as a screening tool for iodine deficiency in humans [36]. Perhaps similar examination could be performed in goats, especially in regions at risk of endemic iodine deficiency.

There are several limitations to this study. First, the effect of the CAE on the thyroid and parathyroid glands cannot be ruled out with certainty. Although the RNA of the virus has been identified in the epithelial cells of the thyroid follicles, anatomopathological lesions are found mainly in the joints, central nervous system, lungs and udder [37]. Therefore, it was assumed that there is no influence of the disease on the morphology and US features of the examined glands. This should be verified in the future studies on goats without CAE. An additional limitation was that goats included in the study belonged to only two breeds. However, both breeds were developed by mating native goats with two popular breeds, i.e. French Alpine (for Polish Fawn Improved) and Saanen (for Polish White Improved) [38]. Considering the above, and in view of the fact that there are relatively small size differences between the most popular goat breeds, the results of this study might also be applied to other breeds, at least until similar research is made for them separately. It should be borne in mind that only female goats were used in the study. Nonetheless, female goats constitute the vast majority of the population in areas where dairy use of goats predominates, and therefore are particularly important for the research.

In conclusion, the normal ultrasonographic characteristics and dimensions of the caprine thyroid and internal parathyroid glands were presented for the first time. The use of thyroid volume formula with a modified correction factor of $\frac{\pi }{4}$ should be considered. Results of the current study may serve as a radiological reference and be the basis for further research.

## Supporting information

S1 Table Ultrasonographic dimensions of the thyroid and parathyroid glands. *l*_*t*_−thyroid length, *w*_*t*_−thyroid width, *h*_*t*_*−*thyroid height, *l*_*p*_−parathyroid length, *w*_*p*_−parathyroid width, R–right lobe, L–left lobe.
(PDF)

S2 Table Gross anatomical dimensions of the thyroid gland. *l*_*t*_−thyroid length, *w*_*t*_−thyroid width, *h*_*t*_*−*thyroid height, *wt*.*–*thyroid weight, R–right lobe, L–left lobe.
(PDF)
