# Supplementary material for: Ultrasonographic and anatomical examination of normal thyroid and internal parathyroid glands in goats
Source: PLoS One. 2020 May 29;15(5):e0233685. doi: 10.1371/journal.pone.0233685 (PMC7259731; doi:10.1371/journal.pone.0233685)
Supplement: S1 Table — lt−thyroid length, wt−thyroid width, ht−thyroid height, lp−parathyroid length, wp−parathyroid width, R–right lobe, L–left lobe. (PDF) [file pone.0233685.s001.pdf]

S1 Table. Ultrasonographic dimensions of the thyroid and parathyroid glands.

l_t – thyroid length, w_t – thyroid width, h_t – thyroid height, l_p – parathyroid length, w_p – parathyroid width, R – right lobe, L – left lobe.

Goat	l_t R [mm]	w_t R [mm]	h_t R [mm]	l_t L [mm]	w_t L [mm]	h_t L [mm]	l_p R [mm]	w_p R [mm]	l_p L [mm]	w_p L [mm]
1	24	12,1	5,7	30	11,7	5,9	2,9	2	3,8	2,2
2	34,8	9,1	6,3	33,7	8,6	5,4	3,1	2,2	3,7	2,2
3	29,4	9,2	6,1	31,7	10,7	6,8	4,3	2,6	3,9	2,6
4	22,1	7,9	5,2	23,3	8,2	5,8	2,5	1,6	2,7	2,1
5	32,1	11,9	4,9	29,8	9,7	5,2	5,4	3,4	4,6	3,2
6	33,6	10,7	7,1	34,8	9,6	6,8	5,6	3,3	4,9	2,9
7	35,1	11	6,4	32,2	10,3	5,5	3,3	2,2	not visible	not visible
8	36,6	10,6	5,3	32,1	13	7,7	3	1,6	3,3	2
9	24,3	10,9	5,2	25,9	10,5	6,5	3,1	2,5	3,4	2,4
10	24,6	7,4	4,4	32,7	7,4	4,7	not visible	not visible	not visible	not visible
11	26,6	13,3	8,7	26,7	13,4	8,1	2,4	2,1	3	2
12	27,7	10,5	5,8	27,8	9,4	5,4	4,3	2,3	3,5	1,9
13	30	8,3	5,4	28,8	8,3	5,3	3,7	2,2	not visible	not visible
14	31,8	10	8,1	30,3	10,8	4,9	1,9	1,6	2	1,5
15	25,1	10,9	6,7	28,7	9,6	6,1	3,6	2,1	4,1	2,6
16	26,4	13	6,5	37,6	7,5	7,4	not visible	not visible	not visible	not visible
17	35,3	14,7	7,3	37,4	13,3	6,4	3,4	2,4	not visible	not visible
18	29,2	9,6	5,2	29	11,8	6	3	1,9	2,5	2
19	28,6	10,9	6	27,2	10,4	7,5	3,9	2,6	3,3	2,2
20	33,1	10,8	7,2	30,4	12,8	6,9	5,3	3,4	5,2	3,2
21	29,5	9,2	6,9	34,5	10	7,8	4	2,8	4,5	3
22	26,1	9,5	4,9	22,8	9	4,9	3,8	2,3	2,5	2
23	29,4	12,4	7,1	26,3	10,2	6,9	4,8	3,5	4,4	2,6
24	26	7	4,2	26,3	8	4,5	3,4	2,3	3,7	1,9
25	25,5	7,9	5	26,8	9,5	4,8	4,2	2,5	3,7	2,6
26	26,3	9	4,7	26,7	9,3	5,1	4,9	3,5	4,4	2,6
27	19,4	8,9	5,2	18	10	6	not visible	not visible	2,5	1,5
28	34,7	14,9	7,8	37,4	13,3	7,4	4,2	2,5	5,3	3,1
29	25,4	12,2	6,5	27,5	11,7	5,5	3,2	2	3,6	2,7
30	26	10,8	6,4	23,5	9,5	4,9	2,5	1,7	2,9	2
31	34,3	11,3	6,2	33,5	10,1	7	4,3	2,6	not visible	not visible
32	28,1	10,4	6,6	29,5	9,9	5,2	not visible	not visible	not visible	not visible
33	3,2	9,9	5,2	30,9	9,4	4,3	4,3	2,9	4,1	3
34	31,2	11,7	6,8	24,1	7	4,3	2,3	1,7	3	2,3
35	28,8	8,5	5,2	27	12,6	7,5	3,5	2,3	not visible	not visible

36	32,5	12,8	7,3	31,5	12,5	8	4,6	2,8	4,4	2,7
37	33,8	10,5	6,5	31,5	8,5	6	4,2	2,6	4	2,5
38	31	10,2	6	32	10	6,5	3,1	1,6	not visible	not visible
39	29,4	11,2	7,6	37,4	12,5	7,3	2,2	1,3	2,9	2
40	31,9	10,4	7,8	29,9	10,1	7	3,4	2,3	3,7	2,2
41	34,1	11,6	6,8	34,3	9,2	7,1	3,9	2,5	4,6	3,6
42	27,8	11,5	5,5	28,5	10,3	5,1	not visible	not visible	not visible	not visible
43	32	10	5,3	34	10,7	6	3,7	1,9	3,9	2,1
44	27,2	8,1	5,1	27,5	8,1	4,5	3	1,9	not visible	not visible
45	34	13,6	9,6	30,1	10,4	6,2	4,5	3,1	not visible	not visible
46	29,1	8,3	4,9	26,4	8,9	4,5	not visible	not visible	not visible	not visible
47	31,1	10,9	6,5	33,3	11	8,2	4,3	2,9	4,8	3,2
48	35,1	14,3	7,9	32,9	13,4	6,8	4,8	2,7	3,9	2,4
49	34,5	9,3	5,3	32,6	9,8	5,8	not visible	not visible	not visible	not visible
50	31	11,5	6,8	27,4	9,6	5,9	4	2,7	3,9	2,6
51	31,8	11,1	5,8	29,2	10,1	4,4	2,3	2,1	2,6	1,6
52	35	9,4	6,1	36,9	12,5	6,6	3,1	2	3,8	2,6
53	35,5	12,1	5,4	31,6	15,5	7,6	3,7	2,9	3,5	2,9
54	31,1	11,1	7,1	31,6	12	6,2	2,4	1,8	2,7	1,7
55	33	9,9	4,9	34	9,6	5,4	5,6	3,6	5	3
56	35	10,8	4,5	33,2	11,4	4,5	4,5	2,8	not visible	not visible
57	31,9	11,9	8,3	32	12,6	6,9	3,8	2,8	3,6	2,4
58	36,4	11,5	7,2	28,2	10,1	7,2	3	2,1	2,7	1,9
59	29,8	10	7,1	27	9,7	6,1	2,8	2	2,7	1,9
60	27,7	10	6,3	28,6	11,7	6,7	3,4	2,1	2,7	2,2
61	33,2	9,5	6,1	31	10,4	6	2,5	1,8	2,7	2,1
62	34,5	10,6	8,1	34,1	11,6	7	not visible	not visible	not visible	not visible
63	28,3	9,4	5,6	29,5	10,5	6,3	4,4	2,8	4,6	2,5
64	26	8,3	5,2	29,7	8,3	5,1	not visible	not visible	2	1,5
65	28,9	11,1	8,1	28	10,2	5,6	3	2,5	not visible	not visible
66	28,1	10,6	6,9	30	9,6	6,3	3,7	2	4	2,8
67	26,3	10,4	7,1	27,6	9	5,4	2	2	3	2
68	29,1	13,1	7,3	27,8	10,1	6,8	4,4	2,5	3,8	2,7
69	29,5	10,5	8,1	32,7	11,7	8,5	2,9	2	3	1,8
70	32,7	11,6	8,8	32,5	12,1	7,5	4,3	3,4	3	2,1
71	32,3	9,5	7,4	30,4	10,2	6,3	3,7	2,9	3,9	2,5
72	32,9	8,1	4,1	33,7	10,6	7	3,9	2,5	4,6	2,8
