# Supplementary material for: Ultrasonographic and anatomical examination of normal thyroid and internal parathyroid glands in goats
Source: PLoS One. 2020 May 29;15(5):e0233685. doi: 10.1371/journal.pone.0233685 (PMC7259731; doi:10.1371/journal.pone.0233685)
Supplement: S2 Table — lt−thyroid length, wt−thyroid width, ht−thyroid height, wt.–thyroid weight, R–right lobe, L–left lobe. (PDF) [file pone.0233685.s002.pdf]

S2 Table. Gross anatomical dimensions of the thyroid gland. l_t – thyroid length, w_t – thyroid width, h_t – thyroid height, *wt.* – thyroid weight, R – right lobe, L – left lobe.

Goat	l_t R [mm]	w_t R [mm]	h_t R [mm]	*wt.* R [g]	l_t L [mm]	w_t L [mm]	h_t L [mm]	*wt.* L [g]
1	31,3	11,5	5,6	1,23	31	12	6	1,47
2	39,9	15,9	8	2,9	37	13,1	7,2	1,98
3	29,6	11,7	6,3	1,22	31,2	12,6	7,1	1,78
4	30	11,5	6,2	1,26	36	10,8	5,9	1,33
5	32,2	12,9	6,3	1,48	40	14,2	7,2	2,3
6	37,5	11,7	7,5	1,82	37	11,9	7,1	1,8
7	41,5	12,2	7,8	2,11	48,5	11,9	6,8	2,14
8	31,5	13,5	8,5	2,23	37,3	14	8,2	2,41
9	26,2	12,5	6	1,18	25,5	11,4	6,2	1,27
10	37,7	9,6	4,7	1,12	20,4	7,7	4,2	0,56
11	28,8	14,8	8,6	2,36	26,4	15	9,3	2,1
12	31,8	11,9	6	1,52	31,8	12,4	5,5	1,44
13	22,2	9,6	6,1	0,71	30,3	10,4	5,6	1,15
14	40,5	12,3	6,2	1,8	40	10,3	5,3	1,4
15	28,5	13,1	7	1,56	31,4	13	6,2	1,56
16	47,7	12,6	5,7	2,1	48,3	12,7	7,1	2,2
17	39,8	16,4	8,7	2,98	34,9	14,5	8,8	2,51
18	31,9	11,4	6,8	1,53	25,3	12,4	5,9	1,3
19	29	12,6	7,5	1,78	28,5	12,7	7,7	1,85
20	35,1	12,9	6,8	1,75	26,5	14,6	8,2	1,67
21	29,6	10,7	7,2	1,29	32	12,4	7	1,64
22	22,2	10,4	5,8	0,97	22,4	10,2	5,4	0,9
23	38	14,5	8,4	2,54	37,5	15,3	8,2	2,53
24	29,2	10,3	5,5	1,01	28,5	10,2	5,9	1,05
25	22,9	9,3	4,9	0,63	22	8,1	5,5	0,57
26	27,4	10,3	6,1	1,63	22,3	9,9	6,2	1,12
27	19,5	10,2	6,2	0,85	19,6	10	7	0,87
28	36,2	17	7,7	3,3	39,1	15,6	8,1	3,42
29	24,3	12,6	7,9	1,47	26,1	12,7	8	1,68
30	25	12,5	6,5	1,36	26,5	13	7,9	1,63
31	32,2	13,9	7,1	1,95	29,7	13,3	7,7	1,99
32	26,4	11,6	6,3	1	24,5	10,9	5,7	0,95
33	27,7	10,4	5,5	0,9	30,5	10,4	5,8	0,97
34	31,5	14,5	7,5	2,18	29,8	13,8	7	1,95
35	27,4	12,9	8,9	1,8	24,6	14,6	7,9	1,61
36	31	16,9	7,5	2,33	41,5	15,9	9,3	3,28
37	36,2	12,5	4,4	1,4	34,8	11,2	4,6	1,15

38	31,5	12	6	1,58	31,5	12,2	5,9	1,53
39	31	12,5	6,6	1,9	35,3	12,7	7,5	2,3
40	30,2	14,8	8	2,2	26,1	12	7,5	1,34
41	33,5	12,2	7,2	1,7	34,2	13,3	6,9	1,85
42	32,2	12,5	6,3	1,32	30,2	11	5,8	1,15
43	27,1	12,1	5,8	1,09	31,3	13,5	6,9	1,84
44	26	9,6	3,9	0,58	27	10,6	4,6	0,89
45	34,2	16,7	10	3,23	32	12,5	6,9	2,5
46	29,7	9,8	4,1	1,71	28,8	13,2	5,8	1,25
47	31,6	12,5	7	1,46	33	16,3	7	2,4
48	35,4	17	8,2	2,79	38,6	17,5	7,5	2,95
49	28,6	9,1	4,2	0,9	32,4	10,5	4,9	1,2
50	27,4	11,7	6,8	1,36	25,5	10,2	5,5	0,9
51	28,2	11,2	6	1,36	37	12	5,4	1,74
52	34	11,2	5,7	1,3	37,2	11,5	8	2,36
53	47,3	15,9	7,5	3,74	43	18,3	9	4
54	30,1	12,8	7	1,6	28,7	12,7	6,3	1,3
55	38,2	13	7	1,97	41,1	13,1	6,8	2,3
56	35,5	12,8	6,5	1,8	32	13,5	6,9	1,9
57	36	13,5	8	2,31	39	16,6	7,8	3,27
58	28,2	15,4	7,4	1,9	23,3	12,5	6,3	1,2
59	31,4	11,7	7,1	1,47	33,3	10,1	5,7	1,29
60	27,4	12,4	6,5	1,4	28	12	6,5	1,4
61	38,8	13,3	6,5	1,82	43,2	12,5	6,8	2,08
62	35,2	14	8,2	2,41	32,6	13	7	1,83
63	31,5	17,3	8	2,8	38,3	11,9	6,4	2,15
64	33	9	4,5	0,8	32,4	9,1	5,4	1,1
65	26,4	13	7,2	1,3	26	11,3	6,3	0,98
66	39,1	17,5	9	3,93	36,2	13,2	8,5	2,37
67	35,2	11,4	7,4	1,53	32,1	10,2	7	1,14
68	35,4	15,1	8	2,63	28,8	14,1	7,2	1,85
69	29,8	12,6	8	2	30,8	15,3	8,3	2,3
70	30,5	12,8	7,3	1,89	31,5	12,4	7,3	1,74
71	28,4	10,8	7,9	1,43	31,1	12,2	8,4	1,81
72	39	11,5	6,9	1,78	37,4	12,6	8,1	2,16
